# Preconception diabetes mellitus and adverse pregnancy outcomes in over 6.4 million women: A population-based cohort study in China

**DOI:** 10.1371/journal.pmed.1002926

**Published:** 2019-10-01

**Authors:** Yumei Wei, Qin Xu, Huixia Yang, Ying Yang, Long Wang, Huan Chen, Craig Anderson, Xinyue Liu, Geng Song, Qian Li, Qiaomei Wang, Haiping Shen, Yiping Zhang, Donghai Yan, Zuoqi Peng, Yuan He, Yuanyuan Wang, Ya Zhang, Hongguang Zhang, Xu Ma

**Affiliations:** 1 Peking University First Hospital, Beijing, China; 2 China DOHaD Research Center, National Human Genetic Resources Center, Beijing, China; 3 National Research Institute for Health and Family Planning, Beijing, China; 4 Graduate School of Peking Union Medical College, Beijing, China; 5 School of Public Health, Institute of Epidemiology and Statistics, Lanzhou University, Lanzhou, China; 6 The George Institute for Global Health (Australia) Beijing Representative Office, Sydney, Australia; 7 Pharmaceutical Outcomes and Policy, College of Pharmacy, University of Florida, Gainesville, Florida, United States of America; 8 Department of Maternal and Child Health, National Health and Family Planning Commission of the PRC, Beijing, China; London School of Hygiene and Tropical Medicine, UNITED KINGDOM

## Abstract

**Background:**

Diabetes mellitus (DM) increases the risk of adverse maternal and neonatal outcomes, and optimization of glycemic control during pregnancy can help mitigate risks associated with diabetes. However, studies seldom focus precisely on maternal blood glucose level prior to pregnancy. We aimed to evaluate the associations between preconception blood fasting plasma glucose (FPG) level and subsequent pregnancy outcomes.

**Methods and findings:**

We conducted a population-based retrospective cohort study among 6,447,339 women aged 20–49 years old who participated in National Free Pre-Pregnancy Checkups Project and completed pregnancy outcomes follow-up between 2010 and 2016 in China. During the preconception health examination, serum FPG concentration was measured, and self-reported history of DM was collected. Women were classified into three groups (normal FPG group: FPG < 5.6 mmol/L and no self-reported history of DM; impaired fasting glucose [IFG]: FPG 5.6–6.9 mmol/L and no self-reported history of DM; and DM: FPG ≥ 7.0 mmol/L or self-reported history of DM). The primary outcomes were adverse pregnancy outcomes, including spontaneous abortion, preterm birth (PTB), macrosomia, small for gestational age infant (SGA), birth defect, and perinatal infant death. Logistic regression model was used to calculate odds ratio (OR) and 95% confidence interval (CI) after adjusting for confounding variables. The mean age of women was 25.24 years, 91.47% were of Han nationality, and 92.85% were from rural areas. The incidence of DM and IFG was 1.18% (76,297) and 13.15% (847,737), respectively. Only 917 (1.20%) women reported a history of DM (awareness of their DM status), of whom 37.28% (337) had an elevated preconception FPG level (≥ 5.6 mmol/L), regarded as noncontrolled DM. A total of 1,005,568 (15.60%) women had adverse pregnancy outcomes. Compared with women with normal FPG, women with IFG had higher risks of spontaneous abortion (OR 1.08; 95% CI 1.06–1.09; *P* < 0.001), PTB (1.02; 1.01–1.03; *P* < 0.001), macrosomia (1.07; 1.06–1.08; *P* < 0.001), SGA (1.06; 1.02–1.10; *P* = 0.007), and perinatal infant death (1.08; 1.03–1.12; *P* < 0.001); the corresponding ORs for women with DM were 1.11 (95% CI 1.07–1.15; *P* < 0.001), 1.17 (1.14–1.20; *P* < 0.001), 1.13 (1.09–1.16; *P* < 0.001), 1.17 (1.04–1.32; *P* = 0.008), and 1.59 (1.44–1.76; *P* < 0.001). Women with DM also had a higher risk of birth defect (OR 1.42; 95% CI 1.15–1.91; *P* = 0.002). Among women without self-reported history of DM, there was a positive linear association between FPG levels and spontaneous abortion, PTB, macrosomia, SGA, and perinatal infant death (*P* for trend <0.001, <0.001, <0.001, 0.001, <0.001). Information about hypoglycemic medication before or during pregnancy was not collected, and we cannot adjust it in the analysis, which could result in underestimation of risks. Data on 2-hour plasma glucose level and HbA1c concentration were not available, and the glycemic control status was evaluated according to FPG value in women with DM.

**Conclusions:**

Women with preconception IFG or DM had higher risk of adverse pregnancy outcomes, including spontaneous abortion, PTB, macrosomia, SGA, and perinatal infant death. Preconception glycemic control through appropriate methods is one of the most important aspects of preconception care and should not be ignored by policy makers.

## Abstract 2

Huixia Yang and colleagues reveal increased risk of adverse pregnancy outcomes for women who have diabetes before pregnancy in a large Chinese cohort.

## Author summary

**Why was this study done?:**

- We found that diabetes mellitus (DM) was associated with increased risk of adverse pregnancy outcomes and that optimization of glycemic control during pregnancy can help mitigate risks associated with diabetes.
- The data of DM among reproductive-aged women planning to conceive remain scarce, particularly in rural China, though they represent about 50% of the Chinese population.
- To our knowledge, this is the first and largest population-based cohort study focusing on the adverse effects of preconception impaired fasting glucose (IFG) or DM on pregnancy outcomes in the population in China.

**What did the researchers do and find?:**

- The awareness rate of DM status among reproductive-age women is extremely low in China, and the management of pregestational DM remains unsatisfactory, even in patients who are aware of their DM status.
- Preconception IFG or DM can increase the risk of adverse pregnancy outcomes, including spontaneous abortion, preterm birth (PTB), macrosomia, small for gestational age infant (SGA), and perinatal infant death.

**What do these findings mean?:**

- Increased screening for DM integrated into primary care should be provided to women who are planning to conceive to improve pregnancy outcomes and promote maternal and neonatal health in future in China.
- It is important to strengthen DM management in Chinese reproductive-aged women.
- Preconception glycemic control through appropriate methods, such as promotion education, hyperglycemia screening and intervening, and high-risk population management, is one of the most important aspects of preconception care and, to improve maternal and neonatal outcomes, should not be ignored by policy makers.

## Introduction

With the aging of the population, urbanization, and related dramatic changes toward sedentary lifestyle during the past few decades, the prevalence of diabetes mellitus (DM) has been growing rapidly worldwide [1–3]. The number of people aged 18 years or older with DM globally is projected to rise from 451 million in 2017 to 693 million in 2045 [4]. In many areas around the world, including the West as well as many developing countries, DM has become a major health burden affecting reproductive-aged women [5–7], which can result in increasing hazards to women’s and children’s health in succession.

In the last 2 decades, with the advance of the developmental origins of health and disease (DOHaD) hypothesis [8], the importance of the adverse impact of maternal hyperglycemia—including DM before pregnancy (preexisting type 1 or type 2 diabetes in pregnancy [1]) and gestational DM (GDM, hyperglycemia occurred during pregnancy)—on pregnancy outcomes and long-term consequences for the offspring has been raised to an unprecedented high level [9,10]. However, it is estimated that almost half of all people (49.7%) living with DM are undiagnosed [4], and among which, many women are unaware of their diabetes status until their first antenatal medical examination. Although preconception counseling was recommended by the Endocrine Society to avoid unintended pregnancy with abnormal glucose level [9], only one-third of women accepted preconception care voluntarily and actively [9,10]. Moreover, hazards to fetus health and pregnancy outcomes caused by hyperglycemia during pregnancy, especially damage of early embryonic development due to DM, cannot be well controlled, even if medical intervention has been implemented since early pregnancy [5].

DM is associated with an increased risk of maternal, perinatal, and neonatal morbidity, which may be similar to those occurring with GDM, but some outcomes (e.g., spontaneous abortions and major malformations) are unique to DM [11]. It has been suggested that appropriate prepregnancy planning is one of the most important steps in reducing the risk of birth defects for women with preexisting diabetes because organogenesis occurs very early in pregnancy [1]. However, studies seldom focus precisely on the association between maternal blood glucose level prior to pregnancy and adverse pregnancy outcomes from a large sample. Sufficient evidence for the association between preconception glucose level as well as DM status and adverse pregnancy outcomes is not only vital for implementation of preconception healthcare strategy including blood glucose screening but also crucial for hyperglycemia management during pregnancy and improving maternal and neonatal outcomes.

Therefore, we conducted a retrospective cohort study among over 6.4 million women aged 20–49 years in China based on the National Free Pre-Pregnancy Checkups Project (NFPCP) to evaluate the associations between preconception serum fasting plasma glucose (FPG) level and subsequent adverse pregnancy outcomes.

## Methods

### Study design and setting

A large population-based retrospective cohort study was conducted among women of reproductive age (20–49 years) who participated in NFPCP from January 1, 2010, to December 31, 2016, successfully became pregnant, and completed pregnancy outcome follow-up before December 31, 2016. NFPCP was initiated by National Health Commission and Ministry of Finance of the People’s Republic of China in 2010 with the aim to provide free preconception health examinations and follow-up of pregnancy outcomes for reproductive-aged couples who planned to conceive within the next 6 months. The project began with serving only rural married couples within 220 counties in 31 provinces from 2010 to 2012 and was further expanded to urban married couples with 2,907 counties in mainland China after 2013. Detailed design, organization, and implementation of this project have been described previously [12–14]. For this study, we did not have a prespecified analysis plan, but we performed hypothesis-driven analyses in which no data-driven changes have taken place. This study is reported per the Strengthening the Reporting of Observational Studies in Epidemiology (STROBE) guideline (S1 STROBE Guideline Checklist). The study was approved by the Institutional Research Review Board at the National Research Institute for Health and Family Planning, Beijing, China. Written informed consent was obtained from all NFPCP participants.

### Participants and recruitment

**Fig 1:**
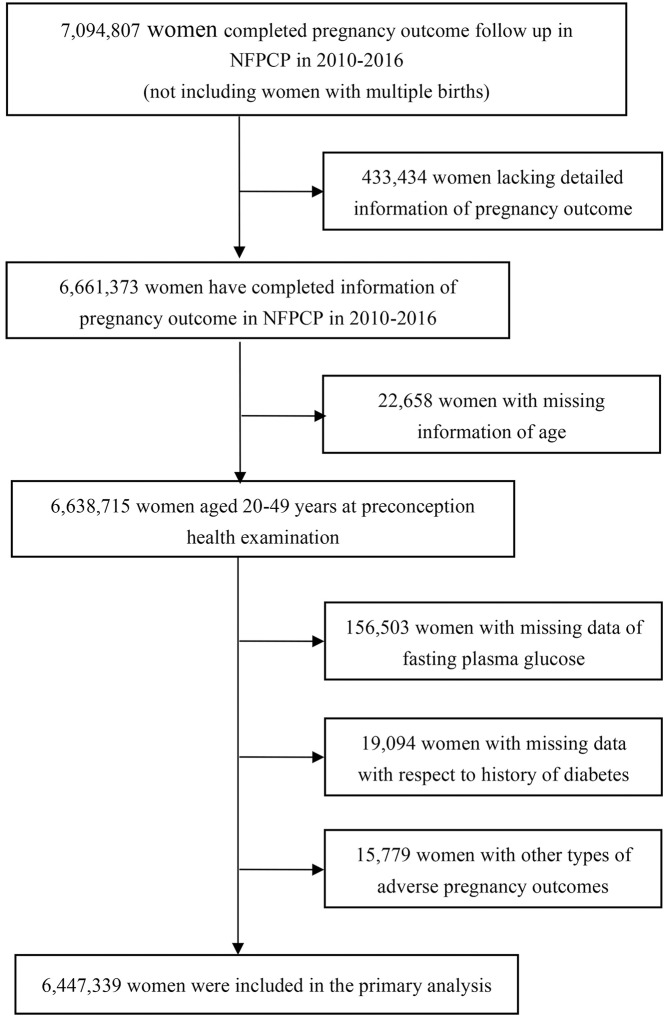
Flowchart of the study population. NFPCP, National Free Pre-Pregnancy Checkups Project.

A total of 6,638,715 women aged 20–49 years old who got pregnant and had completed information of pregnancy outcome from 2010 to 2016 in NFPCP were included in the current study. We then further excluded 156,503 women with missing information on preconception FPG concentration, 19,094 women with missing information with respect to history of diabetes, and 15,779 women who had other types of adverse pregnancy outcomes, such as medically induced abortion, therapeutic induction of labor, and ectopic gestation. As a result, 6,447,339 women were included in the final analysis. Detailed information on the study population recruitment and derivation of the population used in the final analysis are shown in Fig 1.

### Procedures

Briefly, the service includes three stages: preconception health examination, early pregnancy follow-up, and pregnancy outcome follow-up. At the preconception stage, staff from the local resident committee first investigated the willingness of reproductive couples (women aged 20–49 years old, no age limitation was set for men) regarding pregnancy, and then reproductive couples who had already made plans to conceive in the next 6 months were encouraged by staff from the local resident committee to take part in NFPCP. Because NFPCP is a free healthcare program aiming to improve pregnancy outcomes, couples voluntarily came to get the service. Baseline information, including demographic characteristics, lifestyle (self-reported smoking and alcohol consumption), history of chronic diseases (including DM and hypertension), and reproductive history, was collected through a face-to-face interview by trained health staff in the local maternal and child healthcare service centers in each county using a standard and structured questionnaire. Cigarette smoking was defined as smoking at least one cigarette per day for at least 1 year at the time of baseline examination. Then, body weight and height of participants wearing light, indoor clothes and no shoes were measured. Body mass index (BMI) was calculated by dividing the weight in kilograms by the square of the height in meters. Seated blood pressure (BP) was measured in the right arm using an automated BP monitor on a single occasion after participants rested for ≥10 minutes. Hypertension was defined as systolic BP ≥ 140 mmHg or diastolic BP ≥ 90 mmHg or self-reported hypertension. Blood samples after an overnight fast for at least 8 hours were taken and immediately stored at 4–8 °C and then sent to the local laboratories. All data were uploaded and transferred remotely and stored in the NFPCP medical service information system, which was supported by the National Research Institute for Health and Family Planning.

Serum FPG concentration was measured using glucose oxidase or hexokinase methods in the local laboratories in accordance with National Guide to Clinical Laboratory Procedures. The National Center of Clinical Laboratories for Quality Inspection and Detection was responsible for the external quality assessment biannually and for quality control [15]. Women were classified into three groups according to FPG level and self-reported history of DM (normal FPG group: FPG < 5.6 mmol/L and no self-reported history of DM; impaired fasting glucose [IFG]: FPG 5.6–6.9 mmol/L and no self-reported history of DM; and DM: FPG ≥ 7.0 mmol/L or self-reported history of DM) [16]. In the current study, women with self-reported history of DM were defined as the awareness-of-DM-status group, and the other women with DM were defined as the nonawareness group. Among women with self-reported history of DM, women whose FPG level was ≥5.6 mmol/L were considered as the noncontrolled group, and others were the controlled group.

After preconception health examination, all participants were followed up by trained local health workers by telephone. The first interview was conducted within 3 months after baseline examination to track their pregnancy status and record their last menstrual period (LMP). If the participants did not get pregnant at the first interview, repeated inquiries were conducted subsequently within the next 3 months until 1 year after baseline examination. Participants with unsuccessful conception within 1 year after preconception health examination were considered as infertile and were not followed anymore. Participants who had become pregnant were interviewed again for pregnancy outcomes within 1 year after the completion of the first follow-up. Information about the current pregnancy outcomes, delivery date, and neonate conditions was collected.

### Outcomes

The adverse pregnancy outcomes in the current study included (1) preterm birth (PTB), defined as delivery at gestational age between 28 and <37 weeks; (2) macrosomia (newborn birth weight ≥ 4,000 g); (3) small for gestational age infant (SGA, newborn birth weight ≤ 2,500 g of term birth); (4) spontaneous abortion (fetal death occurring before 28 weeks of gestation); (5) birth defects (fetal structural, functional, or metabolic abnormalities that occur before birth, such as anencephaly, hydrocephalus, open spina bifida, cerebrospinal meningitis, cleft lip, cleft palate, congenital heart disease, trisomy 21 syndrome); and (6) prenatal infant death (baby born dead after 28 weeks of gestation or newborns who died after birth within 7 days).

We assessed associations of maternal preconception FPG level with the following primary outcomes: (1) adverse pregnancy outcomes, defined as any of the above adverse pregnancy outcomes; (2) multiple adverse pregnancy outcomes, defined as two or more kinds of adverse pregnancy outcomes; and (3) each of the above adverse pregnancy outcomes. Participants without any adverse pregnancy outcomes of the current pregnancy were identified as controls in data analysis.

### Statistical analysis

Baseline characteristics were presented as means (standard deviation [SD]) for continuous variables and numbers (percentage) for categorical variables. The *χ*^2^ test or analysis of variance (ANOVA) was used to compare the distributions of baseline characteristics among three groups.

To explore the associations between FPG levels and adverse pregnancy outcomes, we calculated the odds ratios (ORs) and their corresponding 95% confidence intervals (CIs) from logistic regression models separately using normal FPG group as the reference group. In order to do a detailed examination of the association between FPG and adverse pregnancy outcomes across a wide range of FPG levels without a priori assumptions about the shape of the dose–response curves, participants without self-reported history of DM were grouped according to FPG in 1 mmol/L categories from <5.0 to >10.0 mmol/L, and then the multivariable-adjusted ORs (95% CIs) were estimated by logistic regression models separately using FPG < 5.0 mmol/L as the reference group. By treating a categorical variable as an ordinal variable in the regression model, we tested the significance of the linear trend. A further analysis was conducted to assess the impact of self-awareness of DM status or glycemic control on adverse pregnancy outcomes among DM participants, using nonawareness group or noncontrolled group as the reference group. All the logistic regressions were adjusted for maternal age at baseline (20–24, 25–29, 30–34, 35–39, and ≥40 years), higher education (defined as senior high school, college or higher; yes/no), area of residence (urban/rural), smoking status (yes/no), alcohol consumption (yes/no), BMI (<18.5, 18.5–23.9, 24.0–27.9, and ≥28.0 kg/m^2^), history of previous adverse pregnancy outcomes (yes/no), hypertension (yes/no), and region of gross domestic product (GDP) per capita (≤10,000, 10,000–20,000, 20,000–30,000, and ≥30,000 Chinese yuan per year).

Sensitivity analyses were conducted by excluding participants with history of adverse pregnancy outcomes. All analyses were performed using R, version 3.2.2, with the “speedglm” packages (https://www.r-project.org/). All statistical tests were two-sided, and values of *P* < 0.05 were considered statistically significant.

## Results

In the current study, a total of 7,094,807 women completed pregnancy outcome follow-up from 2010 to 2016 in the NFPCP, 647,468 participants were then excluded, and 6,447,339 were finally included in the primary analysis (Fig 1). The baseline comparison between included and excluded participants is given in S1 Table. Overall, there were 14.23% (924,034) women with abnormal FPG level: 1.18% (76,297) were DM, and 13.15% (847,737) of women were IFG (Table 1). The baseline characteristics of participants, according to preconception FPG, showed that women with DM or IFG were more likely to have advanced age, higher BMI, less educational attainment, preexisting hypertension, and a history of adverse pregnancy outcomes, including spontaneous abortion, PTB, stillbirth, and birth defects.

**Table 1:** Baseline characteristics of the study population according to preconception FPG.

Characteristics	Total	Groups				Awareness of DM status	Noncontrolled DM
		Normal FPG	IFG	DM	*P*		
***N* (%)**	6,447,339	5,523,305 (85.67)	847,737 (13.15)	76,297 (1.18)		917 (1.20)	347 (37.80)
**FPG (mmol/l, mean [SD])**	4.87 (1.00)	4.64 (0.54)	5.93 (0.31)*	9.18 (5.36)*	<0.001^#^	7.55 (4.09)	11.45 (4.30)
**Age (years, mean [SD])**	25.24 (3.96)	25.18 (3.92)	25.57 (4.16)*	26.04 (4.47)*	<0.001^#^	28.99 (5.03)	29.11 (5.08)
**BMI (kg/m^2^, mean [SD])**	21.21 (2.82)	21.16 (2.78)	21.48 (2.99)*	21.85 (3.50)*	<0.001^#^	24.15 (4.33)	24.50 (4.35)
**Higher education (*n* [%])**	2,253,727 (34.96)	1,948,013 (35.27)	279,587 (32.98)*	26,127 (34.24)*	<0.001	391 (40.46)	129 (37.18)
**Rural inhabitants (*n* [%])**	5,986,668 (92.85)	5,123,581 (92.76)	792,249 (93.46)*	70,838 (92.85)	<0.001	736 (80.26)	63 (18.16)
**Ethnic Han (*n* [%])**	5,897,409 (91.47)	5,050,457 (91.44)	778,680 (91.85)*	68,272 (89.48)*	<0.001	858 (93.57)	322 (92.80)
**History of adverse pregnancy outcomes (*n* [%])**	212,254 (3.29)	179,169 (3.24)	29,895 (3.53)*	3,190 (4.18)*	<0.001	185 (20.17)	65 (18.73)
**History of spontaneous abortion (*n* [%])**	158,702 (2.46)	134,298 (2.43)	22,081 (2.61)*	2,323 (3.05)*	<0.001	101 (11.01)	32 (9.22)
**History of stillbirth (*n* [%])**	43,970 (0.68)	36,901 (0.67)	6,323 (0.75)*	746 (0.98)*	<0.001	77 (8.40)	32 (9.22)
**History of preterm birth (*n* [%])**	10,628 (0.16)	8,862 (0.16)	1,578 (0.19)*	188 (0.25)*	<0.001	13 (1.42)	5 (1.44)
**History of birth defect infant (*n* [%])**	16,547 (0.26)	13,776 (0.25)	2,531 (0.30)*	240 (0.32)*	<0.001	27 (2.94)	7 (2.02)
**Hypertension (*n* [%])**	111,663 (1.73)	90,813 (1.64)	18,505 (2.18)*	2,345 (3.07)*	<0.001	116 (12.65)	53 (15.27)

^#^The analysis of variance was used to examine the differences of baseline characteristics among three groups; others used *χ*^2^ test.

*Multiple comparison with Bonferroni-adjusted *P* value < 0.001, compared with participants with normal FPG.

Abbreviations: BMI, body mass index; DM, diabetes mellitus; FPG, fasting plasma glucose; IFG, impaired fasting glucose; SD, standard deviation

**Fig 2:**
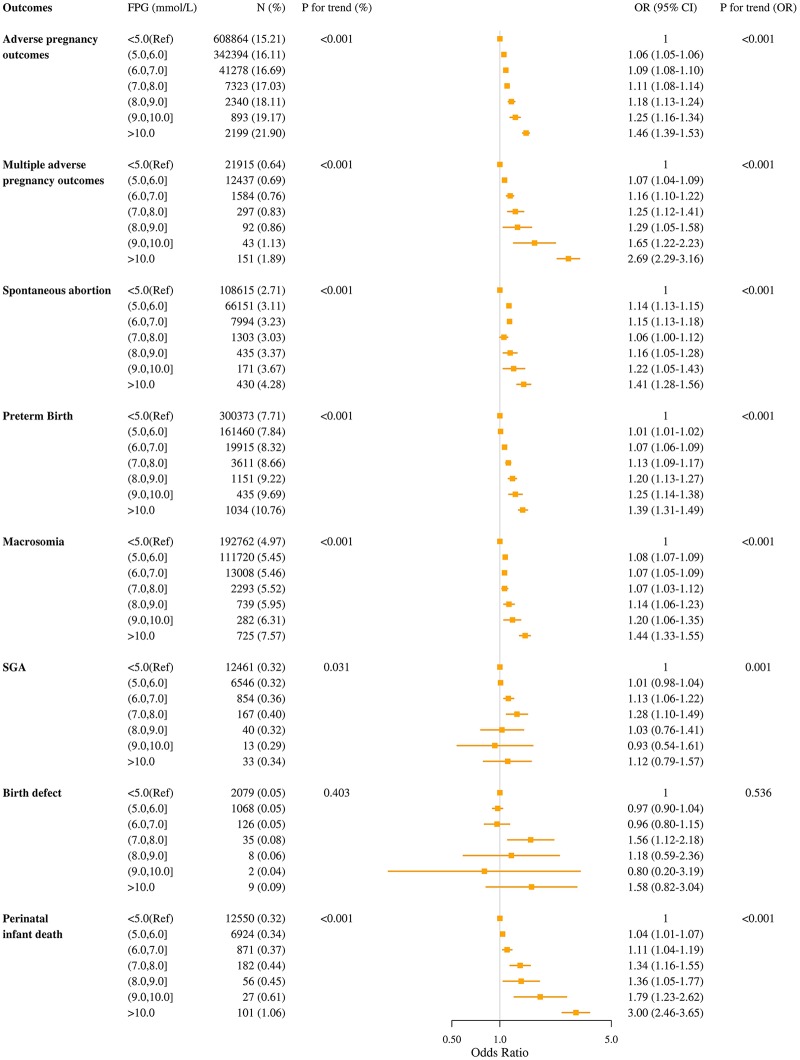
Association between levels of preconception FPG and adverse pregnancy outcomes after excluding participants with self-reported diabetes mellitus. ORs (95% CIs) were adjusted for maternal age at baseline, higher education, area of residence, smoking status, alcohol consumption, body mass index, history of adverse pregnancy outcomes, hypertension, and region of GDP per capita. CI, confidence interval; FPG, fasting plasma glucose; GDP, gross domestic product; OR, odds ratio; Ref, reference group (FPG < 5.0 mmol/L); SGA, small for gestational age infant.

The median length of time from baseline examination to pregnancy was 1.53 months (interquartile range: 0.42–3.96). During the study period, 1,005,568 participants were recorded as having adverse pregnancy outcomes, and the cumulative incidence was 15.60%. The incidence of adverse pregnancy outcomes was 15.46%, 16.29%, and 18.10% for women with normal FPG, IFG, and DM, respectively (Table 2). Compared with women with normal FPG, the multivariable ORs of adverse pregnancy outcomes were 1.05 (95% CI 1.04–1.05; *P* < 0.001) for women with IFG and 1.16 (95% CI 1.14–1.18; *P* < 0.001) for women with DM. The corresponding ORs of multiple adverse pregnancy outcomes were 1.09 (95% CI 1.06–1.12; *P* < 0.001) and 1.44 (95% CI 1.33–1.56; *P* < 0.001), respectively. Among women without self-reported history of DM, there was a positive linear association between FPG levels and adverse pregnancy outcomes (*P* for trend <0.001), and the associations were all statistically significant when FPG level exceeded 5.0 mmol/L (*P* < 0.05) (Fig 2). Similar results were observed in sensitivity analysis after excluding participants with a history of adverse pregnancy outcomes (S2 and S3 Tables).

**Table 2:** Association between preconception FPG and adverse pregnancy outcomes.

Outcomes	Normal FPG (ref)		IFG			DM		
	*n* (%)	OR	*n* (%)	OR (95% CI)	*P* value	*n* (%)	OR (95% CI)	*P* value
Adverse pregnancy outcomes	853,643 (15.46)	1.00	138,119 (16.29)	1.05 (1.04–1.05)	<0.001	13,806 (18.10)	1.16 (1.14–1.18)	<0.001
Multiple adverse pregnancy outcomes	30,711 (0.65)	1.00	5,198 (0.73)	1.09 (1.06–1.12)	<0.001	630 (1.00)	1.44 (1.33–1.56)	<0.001
Spontaneous abortion	156,092 (2.83)	1.00	26,502 (3.13)	1.08 (1.06–1.09)	<0.001	2,563 (3.36)	1.11 (1.07–1.15)	<0.001
Preterm birth	416,153 (7.75)	1.00	65,251 (7.95)	1.02 (1.01–1.03)	<0.001	6,700 (9.09)	1.17 (1.14–1.20)	<0.001
Macrosomia	272,141 (5.09)	1.00	45,106 (5.51)	1.07 (1.06–1.08)	<0.001	4,370 (5.95)	1.13 (1.09–1.16)	<0.001
SGA	17,112 (0.32)	1.00	2,730 (0.33)	1.06 (1.02–1.10)	0.007	277 (0.38)	1.17 (1.04–1.32)	0.008
Birth defect	2,846 (0.05)	1.00	421 (0.05)	0.95 (0.85–1.05)	0.293	62 (0.08)	1.48 (1.15–1.91)	0.002
Perinatal infant death	17,397 (0.33)	1.00	2,927 (0.36)	1.08 (1.03–1.12)	<0.001	401 (0.55)	1.59 (1.44–1.76)	<0.001

Adverse pregnancy outcome indicated accumulated incidences of any adverse pregnancy outcome listed in Table 2. Multiple adverse pregnancy outcome means 2 or more kinds of adverse pregnancy outcomes. ORs (95% CIs) were adjusted for maternal age at baseline, higher education, area of residence, smoking status, alcohol consumption, body mass index, history of adverse pregnancy outcomes, hypertension, and region of GDP per capita.

Abbreviations: CI, confidence interval; DM, diabetes mellitus; FPG, fasting plasma glucose; GDP, gross domestic product; IFG, impaired fasting glucose; OR, odds ratio; ref, reference group (normal FPG); SGA, small for gestational age infant

The cumulative incidences for each of the adverse pregnancy outcomes were as follows: spontaneous abortion (2.88%), PTB (7.79%), macrosomia (5.16%), SGA (0.32%), birth defect (0.05%), and perinatal infant death (0.34%). Women with IFG had significantly higher risk of spontaneous abortion (OR 1.08; 95% CI 1.06–1.09; *P* < 0.001), PTB (1.02; 1.01–1.03; *P* < 0.001), macrosomia (1.07; 1.06–1.08; *P* < 0.001), SGA (1.06; 1.02–1.10; *P* = 0.007), and perinatal infant death (1.08; 1.03–1.12; *P* < 0.001) when compared with those with normal FPG (Table 2). The corresponding ORs for women with DM were 1.11 (95% CI 1.07–1.15; *P* < 0.001), 1.17 (95% CI 1.14–1.20; *P* < 0.001), 1.13 (95% CI 1.09–1.16; *P* < 0.001), 1.17 (95% CI 1.04–1.32; *P* = 0.008), and 1.59 (95% CI 1.44–1.76; *P* < 0.001). Women with DM also had significantly higher risk of birth defect (OR 1.42; 95% CI 1.15–1.91; *P* = 0.002). Linear associations were observed between FPG levels and spontaneous abortion, PTB, macrosomia, SGA, and perinatal infant death (*P* for trend <0.001, <0.001, <0.001, 0.001, <0.001); as the FPG level increased per 1 mmol/L, the risk of spontaneous abortion, PTB, macrosomia, SGA, and perinatal infant death increased 8% (OR 1.08; 95% CI 1.07–1.09), 3% (OR 1.03; 95% CI 1.03–1.04), 5% (OR 1.05; 95% CI 1.05–1.06), 3% (OR 1.03; 95% CI 1.01–1.06), and 9% (OR 1.07; 95% CI 1.07–1.11), respectively, using women with an FPG < 5.0 mmol/L as the reference group (Fig 2).

Among women with DM (76,297), only 1.20% (917) were aware of their DM status, of whom 37.28% (337) had elevated FPG, regarded as noncontrolled DM before pregnancy (Table 3). Compared with women in the nonawareness group, women in the awareness group had significantly higher risk of spontaneous abortion, PTB, macrosomia, and perinatal infant death, with the multivariate ORs of 1.46 (95% CI 1.11–1.92; *P* = 0.007), 1.54 (95% CI 1.27–1.87; *P* < 0.001), 1.40 (95% CI 1.11–1.76; *P* = 0.004), and 2.20 (95% CI 1.27–3.82; *P* = 0.005), respectively. The rates of PTB, macrosomia, and perinatal infant death were significantly lower for women with controlled DM when compared with women with noncontrolled, and the corresponding ORs were 0.38 (95% CI 0.26–0.57; *P* < 0.001), 0.58 (95% CI 0.36–0.92; *P* = 0.020), and 0.08 (95% CI 0.02–0.48; *P* = 0.004), respectively.

**Table 3:** Association between awareness and control of preconception fasting plasma glucose and adverse pregnancy outcomes among participants with DM.

Outcomes	Awareness of DM status							Controlled DM		
	Nonawareness (ref) (*n* = 75,380)		Awareness (*n* = 917)			Noncontrolled (ref) (*n* = 337)		Controlled (*n* = 567)		
	*n* (%)	OR	*n* (%)	OR (95% CI)	*P* value	*n* (%)	OR	*n* (%)	OR (95% CI)	*P* value
Adverse pregnancy outcomes	13,529 (17.95)	1.00	277 (30.21)	1.60 (1.38–1.85)	<0.001	139 (40.06)	1.00	138 (24.21)	0.48 (0.35–0.64)	<0.001
Multiple adverse pregnancy outcomes	610 (0.98)	1.00	20 (3.03)	2.17 (1.36–3.45)	0.001	16 (7.14)	1.00	4 (0.92)	0.11 (0.03–0.35)	<0.001
Spontaneous abortion	2,505 (3.32)	1.00	58 (6.32)	1.46 (1.11–1.92)	0.007	20 (5.76)	1.00	38 (6.67)	1.22 (0.69–2.17)	0.492
Preterm birth	6,574 (9.02)	1.00	125 (14.53)	1.54 (1.27–1.87)	<0.001	71 (21.71)	1.00	54 (10.13)	0.38 (0.26–0.57)	<0.001
Macrosomia	4,282 (5.90)	1.00	88 (10.34)	1.40 (1.11–1.76)	0.004	44 (13.66)	1.00	44 (8.32)	0.58 (0.36–0.92)	0.020
SGA	272 (0.37)	1.00	5 (0.58)	1.53 (0.62–3.77)	0.354	3 (0.92)	1.00	2 (0.38)	0.55 (0.07–4.06)	0.554
Birth defect	60 (0.08)	1.00	2 (0.22)	1.96 (0.46–8.36)	0.365	1 (0.29)	1.00	1 (0.18)	1.00 (0.03–33.42)	0.999
Perinatal infant death	387 (0.54)	1.00	14 (1.65)	2.20 (1.27–3.82)	0.005	12 (3.75)	1.00	2 (0.38)	0.08 (0.02–0.48)	0.004

Adverse pregnancy outcome indicated accumulated incidences of any adverse pregnancy outcome listed in Table 2. Multiple adverse pregnancy outcome means 2 or more kinds of adverse pregnancy outcomes. ORs (95% CIs) were adjusted for maternal age at baseline, higher education, area of residence, smoking status, alcohol consumption, body mass index, history of adverse pregnancy outcomes, hypertension, and region of GDP per capita.

Abbreviations: CI, confidence interval; DM, diabetes mellitus; GDP, gross domestic product; OR, odds ratio; Ref, reference group (nonawareness group or noncontrolled group; SGA, small for gestational age infant

## Discussion

To our knowledge, the present study, conducted in over 6.4 million Chinese reproductive-aged women, is the first large-scale population-based retrospective cohort study to demonstrate the association between preconception FPG level and multiple adverse pregnancy outcomes, taking self-awareness of DM status or glycemic control into consideration. Our study found that women with preconception IFG or DM had higher risk of adverse pregnancy outcomes, including spontaneous abortion, PTB, macrosomia, SGA, and perinatal infant death. A positive linear association was observed between FPG levels and adverse pregnancy outcomes among women without self-reported history of DM. Furthermore, uncontrolled FPG level was significantly associated with higher rate of adverse pregnancy outcomes among women who were aware of their DM status.

Previous studies have shown that hyperglycemia was associated with an increased risk of adverse perinatal outcomes, with hyperglycemia diagnosed during pregnancy rather than before pregnancy [17–19]. Our study confirmed that hyperglycemia before pregnancy was also associated with higher risk of multiple adverse pregnancy outcomes such as spontaneous abortion, PTB, macrosomia, SGA, and perinatal infant death, and DM also had a higher risk of birth defect. These results emphasize the importance of preconception blood glucose test among reproductive-aged women. It would be necessary to establish appropriate policies, such as preconception hyperglycemia screening and high-risk population management, to improve pregnancy outcomes, considering the poor maternal and neonatal health promotion in China, especially in rural areas [20,21].

As estimated by the International Diabetes Federation (IDF), nearly 21.3 million live births (16.2%) were affected by some form of hyperglycemia in pregnancy in 2017 [4], 13.6% of which were due to DM (6.2% was DM detected prior to the pregnancy, and 7.4% was other types of DM detected in pregnancy) [4]. Although the prevalence of DM was very low among reproductive-aged women, the absolute number affected by DM can be large because of the population size in China. Besides, DM has been shown to be related to substantial adverse health outcomes, such as preeclampsia, nephropathy, retinopathy, prematurity, abnormal fetal growth, spontaneous abortion, and congenital malformation [22]. Most of the current guidelines adopt glycosylated hemoglobin (HbA1c < 7.0% or 6.5% without hypoglycemia) to indicate a clinically safe point to satisfy pregnancy outcomes for women with DM or elevated blood glucose [23–25]. Our study showed a positive and significant linear relationship between FPG levels and adverse pregnancy outcomes including spontaneous abortion, PTB, macrosomia, and perinatal infant death among women without self-reported history of DM when compared with preconception FPG < 5.0 mmol/L, indicating a relatively safe FPG value for women planning to conceive in terms of preventing adverse pregnancy outcomes. As the FPG level increased per 1 mmol/L, the risk of spontaneous abortion, PTB, macrosomia, SGA, and perinatal infant death increased 8%, 3%, 5%, 3%, and 9%, respectively, using women with FPG < 5.0 mmol/L as the reference group; FPG should be used as an important evaluation indicator of preconception glycemic control when HbA1c is absent in low-resource areas.

Our study found that the awareness rate of DM status was only 1.20% among reproductive-aged women in China. Low awareness rate can be the result of limited knowledge on DM due to lower socioeconomic development status and unevenly distributed health service resources in China. This may also lead to increased risk of uncontrolled blood glucose level, which has been proved to be associated with higher risk of adverse pregnancy outcomes in our study.

Given the large overall and rural population in China, high percentage of misdiagnosis of diabetes, low proportion of people receiving DM treatment, and low proportion of Chinese women reaching target blood glucose level [26], the low awareness rate of DM status may result in a severe challenge to management of DM and improvement of adverse pregnancy outcomes in China. Meanwhile, the examination and treatment of DM for reproductive-aged women planning to conceive should be covered by medical insurance in China. Policy decisions on adoption of an extensive screening of DM in prepregnancy examination and strategies for DM and adverse pregnancy outcomes control from a broader perspective require more evidence on the cost-effectiveness of such approaches. Women with DM should be referred to tertiary hospitals or DM centers for further evaluation and treatment before and after pregnancy, and hierarchical diagnosis and treatment systems for diabetes control in pregnant women should be implemented.

Interestingly, our study found that women who were aware of their DM status had higher risk of adverse pregnancy outcomes. This result could be affected by some confounders, such as length of disease and severity of complications among women with DM; however, we were not able to adjust them, because of data availability. Thus, further study to confirm the association between level of awareness and adverse pregnancy outcomes, as well as cost-effectiveness of extensive examination of FPG for women who are planning to conceive, is still needed before any decision can be made from the public health policy perspective.

The result from the present analysis showed that the prevalence of DM before pregnancy was 1.18% in China (most of them from rural areas), which was much lower than the results from a nationally cross-sectional survey in 2010 that reported the prevalence of DM was 4.5%, 6.6%, and 11.3% in Chinese women aged 18–29, 30–39, and 40–49 years, respectively [26]. There are several reasons contributing to the difference: first, the women included in this study were those with a plan to conceive within 6 months and were likely to be healthier; second, women who did not conceive within 1 year after the baseline examination were not followed anymore, which could result in bias; third, the high incidence of misdiagnosis of DM (up to 69.4%) among Chinese women can also lead to a reduced prevalence of DM [26]; finally, we defined DM as a self-reported history of DM or FPG ≥ 7.0 mmol/L, not including 2-hour plasma glucose level ≥ 11.1 mmol/L, or HbA1c concentration of ≥6.5% because of the data availability [26], so our results may be underestimated.

To our knowledge, this is the first and largest nationwide coverage cohort study to investigate the association between preconception FPG level and substantial adverse pregnancy outcomes and self-awareness of DM or glycemic control with outcomes. Detailed information regarding lifestyle (smoking and drinking), history of previous adverse pregnancy outcomes, disease history (hypertension), and other important confounding variables, such as maternal age and economic level (region of GDP per capita), were all controlled in the current study. Thus, the relationship between maternal preconception FPG levels and adverse pregnancy outcomes can be well evaluated. However, some limitations should be mentioned. First, not all of the reproductive-aged women who plan to conceive in China participated in the NFPCP, and most of the participants in our study were from rural areas of China (92.85%). This may lead to an underestimated result of the DM prevalence and awareness rate. Secondly, oral glucose tolerance test, HbA1c, and postprandial blood glucose concentrations were not measured; thus, the prevalence of IFG or DM could be underestimated. Third, data on 2-hour plasma glucose level and HbA1c concentration were not available, and the glycemic control status was evaluated according to FPG value in women with DM. Fourth, data on other adverse complications relevant to high blood glucose, such as shoulder dystocia, neonatal hypoglycemia, etc., were not collected and limited our analysis. Finally, NFPCP provides preconception health counseling according to the examination results. It was recommended for hyperglycemic women to go to the hospital for treatment. However, we did not collect information about the hypoglycemic medication used before or during pregnancy, which would result in underestimation of the risks.

### Conclusions

In summary, our study found that women with preconception IFG or DM had higher risk of adverse pregnancy outcomes, including spontaneous abortion, PTB, macrosomia, SGA, and perinatal infant death. The awareness rate of DM status among reproductive-aged women is extremely low, and the management of DM remains unsatisfactory, even in patients who are aware of their DM status. Preconception glycemic control through appropriate methods, such as promotion education, hyperglycemia screening and intervening, and high-risk population management, is one of the most important aspects of preconception care and, to improve maternal and neonatal outcomes, should not be ignored by policy makers.

## Supporting information

S1 STROBE Guideline Checklist STROBE, Strengthening the Reporting of Observational Studies in Epidemiology. (DOC)

S1 Table Comparisons of baseline characteristics between included and excluded participants. (DOCX)

S2 Table Sensitivity analysis of association between preconception FPG and adverse pregnancy outcomes after excluding participants with history of adverse pregnancy outcomes. FPG, fasting plasma glucose.
(DOCX)

S3 Table Sensitivity analysis of association between levels of preconception FPG and pregnancy outcomes after excluding participants with self-reported DM and history of adverse pregnancy outcomes. DM, diabetes mellitus; FPG, fasting plasma glucose.
(DOCX)

## Abbreviations

ANOVA: analysis of variance
BMI: body mass index
BP: blood pressure
CI: confidence interval
DM: diabetes mellitus
DOHaD: developmental origins of health and disease
FPG: fasting plasma glucose
GDM: gestational DM
GDP: gross domestic product
IDF: International Diabetes Federation
IFG: impaired fasting glucose
LMP: last menstrual period
NFPCP: National Free Pre-Pregnancy Checkups Project
OR: odds ratio
PTB: preterm birth
SD: standard deviation
SGA: small for gestational age infant
STROBE: Strengthening the Reporting of Observational Studies in Epidemiology
