# Supplementary material for: Preconception diabetes mellitus and adverse pregnancy outcomes in over 6.4 million women: A population-based cohort study in China
Source: PLoS Med. 2019 Oct 1;16(10):e1002926. doi: 10.1371/journal.pmed.1002926 (PMC6771981; doi:10.1371/journal.pmed.1002926)
Supplement: S1 Table — (DOCX) [file pmed.1002926.s002.docx]

**S1 Table. Comparisons of baseline characteristics between included and excluded participants.**

| Characteristics | Excluded  (N=647468) | Included  (N=6447339) | *P* |
| --- | --- | --- | --- |
| FPG (mmol/l, mean [SD]) | 4.87 (1.04) | 4.87 (1.00) | 0.008**^#^** |
| Age (years, mean [SD]) | 25.54 (4.25) | 25.24 (3.96) | <0.001**^#^** |
| BMI (kg/m^2^, mean [SD]) | 21.20 (2.84) | 21.21 (2.83) | 0.402**^#^** |
| Higher education (n [%]) | 244670 (37.79) | 2253727 (34.96) | <0.001 |
| Rural inhabitants (n [%]) | 577585 (89.21) | 5986668 (92.86) | <0.001 |
| Ethnic Han (n [%]) | 569342 (87.93) | 6447339 (91.47) | <0.001 |
| History of adverse pregnancy outcomes (n [%]) | 17751 (2.90) | 212254 (3.29) | <0.001 |
| History of spontaneous abortion (n [%]) | 14000 (2.16) | 158702 (2.46) | <0.001 |
| History of stillbirth (n [%]) | 3761 (0.58) | 43970 (0.68) | <0.001 |
| History of PTB (n [%]) | 1095 (0.17) | 10628 (0.17) | 0.999 |
| History of birth defect infant (n [%]) | 1588 (0.25) | 16547 (0.26) | 0.130 |
| Hypertension (n [%]) | 11699 (1.81) | 111663 (1.73) | <0.001 |

FPG, fasting plasma glucose; SD, standard deviation; BMI, body mass index.

**^#^** The t test was used to examine the differences of baseline characteristics among three groups, others used $\chi^{2}$ test.
