# Supplementary material for: Preconception diabetes mellitus and adverse pregnancy outcomes in over 6.4 million women: A population-based cohort study in China
Source: PLoS Med. 2019 Oct 1;16(10):e1002926. doi: 10.1371/journal.pmed.1002926 (PMC6771981; doi:10.1371/journal.pmed.1002926)
Supplement: S2 Table — FPG, fasting plasma glucose. (DOCX) [file pmed.1002926.s003.docx]

**S2 Table. Sensitivity analysis of association between preconception FPG and adverse pregnancy outcomes after excluding participants with history of adverse pregnancy outcomes.**

| **Outcomes** | **normal FPG (ref)** | |  | **IFG** | |  | **DM** | |
| --- | --- | --- | --- | --- | --- | --- | --- | --- |
|  | n (%) | OR |  | n (%) | OR (95% CI) |  | n (%) | OR (95% CI) |
| Adverse pregnancy outcomes | 818139 (15.35) | 1.00 |  | 131622 (16.14) | 1.05 (1.04-1.05) |  | 13036 (17.89) | 1.16 (1.14-1.18) |
| Multiple adverse pregnancy outcomes | 29155 (0.64) | 1.00 |  | 4900 (0.71) | 1.08 (1.05-1.12) |  | 580 (0.96) | 1.43 (1.31-1.55) |
| Spontaneous abortion | 147492 (2.77) | 1.00 |  | 24908 (3.05) | 1.08 (1.06-1.09) |  | 2381 (3.27) | 1.11 (1.07-1.16) |
| Preterm birth | 399989 (7.72) | 1.00 |  | 62359 (7.88) | 1.02 (1.01-1.03) |  | 6357 (9.02) | 1.17 (1.14-1.20) |
| Macrosomia | 262115 (5.08) | 1.00 |  | 43170 (5.48) | 1.06 (1.05-1.07) |  | 4132 (5.89) | 1.12 (1.09-1.16) |
| SGA | 16203 (0.31) | 1.00 |  | 2577 (0.33) | 1.06 (1.02-1.10) |  | 268 (0.38) | 1.22 (1.08-1.38) |
| Birth defect | 2640 (0.05) | 1.00 |  | 392 (0.05) | 0.96 (0.86-1.06) |  | 55 (0.08) | 1.45 (1.11-1.89) |
| Perinatal infant death | 16389 (0.32) | 1.00 |  | 2752 (0.35) | 1.08 (1.04-1.12) |  | 367 (0.52) | 1.58 (1.42-1.75) |

Adverse pregnancy outcome indicated accumulated incidences of any adverse pregnancy outcome listed in S2 Table. Multiple adverse pregnancy outcome means 2 or more kinds of adverse pregnancy outcomes. ORs (95% CIs) were adjusted for maternal age at baseline, higher education, area of residence, smoking status, alcohol consumption, body mass index, history of adverse pregnancy outcomes, hypertension, and region of GDP per capita.

Abbreviations: CI, confidence interval; DM, diabetes mellitus; FPG, fasting plasma glucose; GDP, gross domestic product; IFG, impaired fasting glucose; OR, odds ratio; ref, normal FPG was used as the reference group in the model; SGA, small for gestational age infant
