# Supplementary material for: Preconception diabetes mellitus and adverse pregnancy outcomes in over 6.4 million women: A population-based cohort study in China
Source: PLoS Med. 2019 Oct 1;16(10):e1002926. doi: 10.1371/journal.pmed.1002926 (PMC6771981; doi:10.1371/journal.pmed.1002926)
Supplement: S3 Table — DM, diabetes mellitus; FPG, fasting plasma glucose. (DOCX) [file pmed.1002926.s004.docx]

**S3 Table. Sensitivity analysis of association between levels of preconception FPG and pregnancy outcomes, after excluding participants with self-reported DM and history of adverse pregnancy outcomes.**

| **Outcomes** | | **<5.0(Ref)** | **(5.0,6.0]** | **(6.0,7.0]** | **(7.0,8.0]** | **(8.0,9.0]** | **(9.0,10.0]** | **>10.0** | **P for trend** |
| --- | --- | --- | --- | --- | --- | --- | --- | --- | --- |
| Adverse pregnancy outcomes | N (%) | 584084 (15.11) | 327205 (15.98) | 39215 (16.52) | 6971 (16.9) | 2220 (17.95) | 834 (18.8) | 2057 (21.56) | <0.001 |
|  | OR (95% CI) | 1.00 | 1.06 (1.05-1.06) | 1.09 (1.08-1.1) | 1.12 (1.09-1.14) | 1.19 (1.13-1.24) | 1.24 (1.15-1.33) | 1.45 (1.38-1.52) | 0.002 |
| Multiple adverse pregnancy outcomes | N (%) | 20839 (0.63) | 11758 (0.68) | 1484 (0.74) | 275 (0.8) | 87 (0.85) | 40 (1.10) | 140 (1.84) | <0.001 |
|  | OR (95% CI) | 1.00 | 1.06 (1.04-1.09) | 1.15 (1.09-1.21) | 1.24 (1.10-1.39) | 1.30 (1.05-1.61) | 1.65 (1.21-2.26) | 2.69 (2.28-3.18) | 0.019 |
| Spontaneous abortion | N (%) | 102681 (2.66) | 62421 (3.05) | 7461 (3.14) | 1227 (2.98) | 405 (3.27) | 156 (3.52) | 386 (4.05) | 0.018 |
|  | OR (95% CI) | 1.00 | 1.14 (1.13-1.15) | 1.15 (1.12-1.18) | 1.07 (1.01-1.13) | 1.16 (1.05-1.28) | 1.21 (1.03-1.42) | 1.38 (1.24-1.53) | <0.001 |
| Preterm Birth | N (%) | 289005 (7.68) | 154669 (7.79) | 19005 (8.26) | 3458 (8.64) | 1093 (9.13) | 405 (9.46) | 977 (10.67) | <0.001 |
|  | OR (95% CI) | 1.00 | 1.01 (1.00-1.02) | 1.07 (1.06-1.09) | 1.13 (1.09-1.17) | 1.20 (1.12-1.27) | 1.23 (1.11-1.37) | 1.40 (1.31-1.49) | <0.001 |
| Macrosomia | N (%) | 185833 (4.96) | 107263 (5.42) | 12413 (5.42) | 2 176 (5.46) | 706 (5.93) | 269 (6.32) | 687 (7.54) | <0.001 |
|  | OR (95% CI) | 1.00 | 1.08 (1.07-1.09) | 1.07 (1.05-1.09) | 1.06 (1.02-1.11) | 1.14 (1.06-1.23) | 1.21 (1.07-1.37) | 1.44 (1.33-1.56) | 0.011 |
| SGA | N (%) | 11808 (0.31) | 6184 (0.31) | 806 (0.35) | 161 (0.4) | 39 (0.33) | 13 (0.30) | 32 (0.35) | 0.017 |
|  | OR (95% CI) | 1.00 | 1.01 (0.98-1.04) | 1.14 (1.06-1.22) | 1.31 (1.12-1.54) | 1.07 (0.78-1.47) | 1 (0.58-1.73) | 1.17 (0.83-1.66) | 0.527 |
| Birth defect | N (%) | 1935 (0.05) | 986 (0.05) | 117 (0.05) | 30 (0.07) | 8 (0.06) | 2 (0.05) | 9 (0.09) | 0.444 |
|  | OR (95% CI) | 1.00 | 0.96 (0.89-1.04) | 0.97 (0.81-1.17) | 1.45 (1.01-2.09) | 1.29 (0.64-2.58) | 0.88 (0.22-3.52) | 1.75 (0.91-3.37) | 0.178 |
| Perinatal infant death | N (%) | 11845 (0.32) | 6513 (0.33) | 804 (0.35) | 170 (0.43) | 53 (0.45) | 24 (0.57) | 90 (0.99) | <0.001 |
|  | OR (95% CI) | 1.00 | 1.04 (1.01-1.07) | 1.10 (1.02-1.18) | 1.34 (1.16-1.57) | 1.39 (1.06-1.82) | 1.73 (1.16-2.59) | 2.91 (2.36-3.59) | 0.015 |

Adverse pregnancy outcome indicated accumulated incidences of any adverse pregnancy outcome listed in S3 Table. Multiple adverse pregnancy outcome means 2 or more kinds of adverse pregnancy outcomes. ORs (95% CIs) were adjusted for maternal age at baseline, higher education, area of residence, smoking status, alcohol consumption, body mass index, history of adverse pregnancy outcomes, hypertension, and region of GDP per capita.

Abbreviations: CI, confidence interval; DM, diabetes mellitus; FPG, fasting plasma glucose; GDP, gross domestic product; OR, odds ratio; Ref, FPG < 5.0 mmol/L was used as the reference group in the model; SGA, small for gestational age infant.
